# Metastasis Extraction From Non–Small Cell Lung Cancer Clinical Notes Using Large Language Model–Based Classification: Retrospective Comparative Study

**DOI:** 10.2196/99573

**Published:** 2026-10-16

**Authors:** Sweta Balaji, Kiersten Campbell, Rou-Zhen Chen, Daniel Smith, Matthew A Reyna, Abeed Sarker, Joshua D Wallach, Ravi B Parikh, Selen Bozkurt

**Affiliations:** 1 Department of Biomedical Informatics School of Medicine Emory University Atlanta, GA United States; 2 Department of Epidemiology Rollins School of Public Health Emory University Atlanta, GA United States; 3 Department of IT and Research Informatics Winship Cancer Institute Atlanta, GA United States; 4 Department of Hematology and Medical Oncology School of Medicine Emory University Atlanta, GA United States

**Keywords:** electronic health records, large language models, metastasis, natural language processing, non-small-cell lung cancer

## Abstract

**Background:**

Identification of metastasis status in non–small cell lung cancer (NSCLC) is a critical part of understanding disease prognosis, treatment courses, trial eligibility, and population-level cancer surveillance. However, metastasis status is inconsistently recorded in structured cancer registry fields, since manual abstraction of clinical notes is often an error-prone process. This challenge highlights an opportunity for leveraging large language models (LLMs) to conduct high-scale metastasis extraction from real-world clinical documentation.

**Objective:**

We conducted a retrospective, multicohort comparative evaluation of 3 distinct LLMs for 2 independent classification tasks: overall metastasis presence at any site and brain/central nervous system (CNS) metastasis presence. We evaluated model performance on 2 independent NSCLC cohorts: one with registry labels and another with manual annotations. We further explored whether our methods could analyze clinical documentation and recover missing information in structured registry fields.

**Methods:**

Patient cohorts were derived from the Winship Cancer Institute. Cohort 1 (n=579 patients; 24,887 notes across 69 note types; 2023-2025) used registry-linked metastasis fields as the reference standard. Cohort 2 (n=22 patients; 644 radiology notes; 2010-2021) was drawn from 2 completed randomized trials and used dual-annotated labels (Cohen κ: 0.93 overall metastasis; 0.88 CNS metastasis) as the reference standard. We fine-tuned the GatorTron-base encoder model for each independent binary classification task. We evaluated MedGemma-27B-text and Llama 3.1-70B using zero-shot prompting. A separate cohort of 675 patients with missing registry labels was used for an exploratory missingness-recovery analysis, validated against annotations of a random subsample. Our best-performing model was used to conduct further site-specific metastasis classification, based on available registry data.

**Results:**

More than half (675/1260, 54%) of initially identified cohort 1 patients had missing or unknown registry metastasis labels. MedGemma demonstrated the best performance in overall metastasis classification (cohort 1: *F*_1_-score=0.80; cohort 2 patient-level: *F*_1_-score=1.0; cohort 2 note-level: *F*_1_-score=0.93). For brain/CNS metastasis, Llama3 performed best in both cohorts (cohort 1: *F*_1_-score=0.79; cohort 2 patient-level: *F*_1_-score=0.93; cohort 2 note-level: *F*_1_-score=0.86). The fine-tuned GatorTron model showed strong performance for classification of overall metastasis in cohort 1 (*F*_1_-score=0.72). Error analysis indicated that several model errors reflected incomplete registry labels, ambiguous clinical language, or missing documentation. In the exploratory recovery analysis, model predictions agreed with manual annotations at an accuracy of 0.90 and *F*_1_-score of 0.89. Additional site-specific metastasis classification by MedGemma demonstrated high performance for liver metastasis (*F*_1_-score=0.82) and bone metastasis (*F*_1_-score=0.79).

**Conclusions:**

All models demonstrated relatively high performance. The zero-shot generative models were more robust to nuanced documentation and context-dependent brain/CNS metastasis extraction. The fine-tuned encoder model demonstrated strong classification performance but may have been limited by potential inaccuracies in the registry reference standards during model training. This study further demonstrated the potential of LLMs in recovering plausible structured labels from narrative text, complementing cancer registries for metastasis ascertainment.

## Introduction

Non–small cell lung cancer (NSCLC) is the leading cause of cancer-related mortality worldwide, with prognosis and treatment shaped by the presence and extent of metastatic disease at diagnosis and throughout the clinical course [1-3]. Among the most consequential and clinically complex sites of spread is the central nervous system (CNS) [4]. Brain metastasis develops in up to 50% of patients with NSCLC over the course of their disease, carrying distinct prognostic implications, driving treatment decisions, and serving as a key eligibility criterion in oncology trials [5,6]. Accurate ascertainment of metastatic status is crucial for determining eligibility for systemic therapies, radiation planning, enrollment in clinical trials, and cancer surveillance [7,8]. These realities underscore that metastasis information, particularly CNS metastasis, must be captured accurately and comprehensively.

However, metastatic status is inconsistently recorded in structured electronic health record (EHR) fields and is often dispersed across radiology reports, oncology progress notes, discharge documentation, and other free-text clinical narratives [9]. Cancer registries, the primary infrastructure for population-level cancer surveillance, rely on manual review by trained cancer registrars, an approach that is resource-intensive, subject to human error, and limited in its ability to capture disease progression documented after the initial abstraction window [10]. These gaps in data capture can have meaningful consequences. In a large National Cancer Database analysis of more than 1.2 million patients with NSCLC, 71% had missing data in at least one variable of interest, including cancer stage and treatment [11]. Missing data were especially prevalent among patients with lower 2-year overall survival, indicating that incomplete documentation may have been more prevalent for patients with severe illnesses or complex, metastatic cases [11]. Thus, studies using cancer registry data may be biased if missingness is ignored [11].

Another limitation of cancer registries is that data often reflect disease status primarily at the time of initial diagnosis and do not capture metastatic progression documented later in the clinical course [12,13]. In contrast, clinical notes provide longitudinally detailed records of disease evolution, including imaging interpretation, treatment modifications, and clinician assessments across the patient journey. Manual abstraction of clinical notes is a lengthy, resource-intensive process that is prone to human error and may not comprehensively capture disease complexities documented throughout the course of a patient’s medical history [14]. These challenges highlight an opportunity for informatics approaches, namely natural language processing (NLP) methods, to enable large-scale extraction of metastasis records from real-world clinical documentation, improving accuracy and comprehensiveness of existing structured data sources [15].

There has been rapid evolution of NLP methods in the field of clinical information extraction. Early rule-based methods leveraged medical ontologies to create structured rules for detection of relevant information in unstructured text [16]. This method continues to be used today, particularly for text filtering and standardization [17]. Rule-based methods have previously demonstrated high precision and interpretability but with limited ease of implementation and generalizability to notes beyond the study context [18,19]. Their limited generalizability is characterized by their dependence on specific linguistic patterns, along with requiring specialized human expertise to create custom rules [20]. These rules can be affected by the unique documentation style and phrasing of the data used [20]. Machine learning (ML) approaches demonstrated improvement over rule-based systems due to their ability to automatically learn linguistic patterns and complexities rather than solely capturing patterns based on predefined rules [21]. Transformer-based ML models, specifically, have demonstrated strong performance in real-world data and can be fine-tuned for use in various clinical contexts [21]. Transformers pretrained on clinical and medical data have further demonstrated immense promise in detecting semantic interactions and complex, varied phrasing in clinical text [22,23]. These models, further optimized on smaller clinical cohorts, have been shown to outperform traditional NLP and ML approaches, including regular expression-based text searches and classic supervised learning approaches [24]. However, lower performance has been observed when applying models, including transformer-based, rule-based, and classic ML methods, to heterogeneous note types or switching institutions from which the notes are sourced [21,23]. Furthermore, differences in vocabulary and documentation style can be seen to lower performance in these models [21,23]. More recently, generative large language models (LLMs), including both domain-adapted and general-purpose instruction-tuned variants, have demonstrated strong performance on a range of clinical extraction tasks [11,25-27]. However, because generative LLMs produce free, unconstrained text rather than directly predicting predefined labels, additional prompting and/or postprocessing may be required to standardize their outputs [28]. These additional steps may introduce variability in output formatting and interpretation, whereas BERT-based classifiers map inputs directly to a fixed label set [28]. These models also require a substantial amount of memory, which can detract from widespread use in clinical settings [23].

Despite this progress, critical gaps remain, particularly in the types of clinical text analyzed for extraction tasks. First, a recent systematic review of LLM applications in clinical medicine found that over three-quarters of included studies did not conduct analyses on real EHR data, relying instead on simulated clinical scenarios or synthetic notes [29]. This limits the generalizability of findings to the nuanced documentation encountered in routine oncology practice. Second, few studies have directly compared fine-tuned domain-adapted encoder models, for supervised classification, against instruction-tuned generative models, particularly for information extraction from real-world oncology notes [23,28]. Third, and particularly for NSCLC, brain and CNS metastasis extraction is underexamined as a distinct NLP task, despite its clinical importance. Given the complex language and the plethora of terminologies used to document different sites of CNS metastasis, this extraction task further tests the ability of NLP methods or LLMs in detecting and distinguishing between nuanced phrases and site-specific verbiage.

To address these gaps, we examined 2 research questions. First, how accurately can LLM-based methods classify the presence of overall metastasis and site of metastasis from real-world clinical notes? Second, can these models recover clinically relevant metastasis information that is missing from structured registry data? Metastasis status recorded in structured registry fields is a crucial part of observational cancer research and trial recruitment, so systematic undercapture propagates into every downstream use. A pipeline addressing this gap would operate retrospectively on documentation already in the EHR. In cancer registry abstraction, where metastasis status is currently determined by manual chart review, it would prescreen notes and help identify candidate documentation for abstractors. In trial eligibility screening, it would flag patients whose metastasis status is absent or incomplete in structured fields and who may be misclassified during cohort identification. In both cases, the model narrows what a human reviews rather than deciding the classification.

## Methods

### Study Design and Setting

We conducted a retrospective comparative evaluation of 3 LLM-based methods for extracting (1) overall metastasis at any site and (2) brain/CNS metastasis from real-world clinical notes in patients with NSCLC. We leveraged 2 cohorts with distinct but complementary roles in model development and validation, in addition to a dataset with missing metastasis records to explore the LLM’s capabilities for data recovery. All analyses were conducted under a waived-consent protocol approved by the Emory University Institutional Review Board.

### Data Sources and Cohorts

#### Cohort 1: Primary Registry-Linked Development Cohort

##### Overview

Cohort 1 was used for model development and internal validation. It was derived from the Winship Cancer Registry, which is linked to the Georgia Comprehensive Cancer Registry. Inclusion was restricted to patients for whom Winship Cancer Institute was the institution of record for diagnosis and/or first-course treatment, with both registry data and linked clinical notes available from 2023 through 2025.

##### Cohort Construction and Exclusions

Initial data extraction yielded 1260 patients with NSCLC with 62,191 notes across 81 note types and 12,174 encounters. Patients were excluded from model development if at least one registry metastasis field was missing (n=663) or labeled unknown with no confirmed positive site (n=12), yielding 585 patients with 24,908 notes across 69 note types. Six additional patients were excluded from model development and validation because their available notes contained no cancer-relevant clinical content (eg, administrative telephone encounters only), yielding a final development cohort of 579 patients with 24,887 notes across 69 note types for overall metastasis analysis. Three additional patients with unconfirmed brain metastasis registry records were excluded from the brain/CNS metastasis analysis, yielding a cohort of 576 patients. The full cohort selection process is illustrated in Figure 1. Demographic characteristics, following initial data extraction and cohort selection, are described in Table 1.

**Figure 1:**
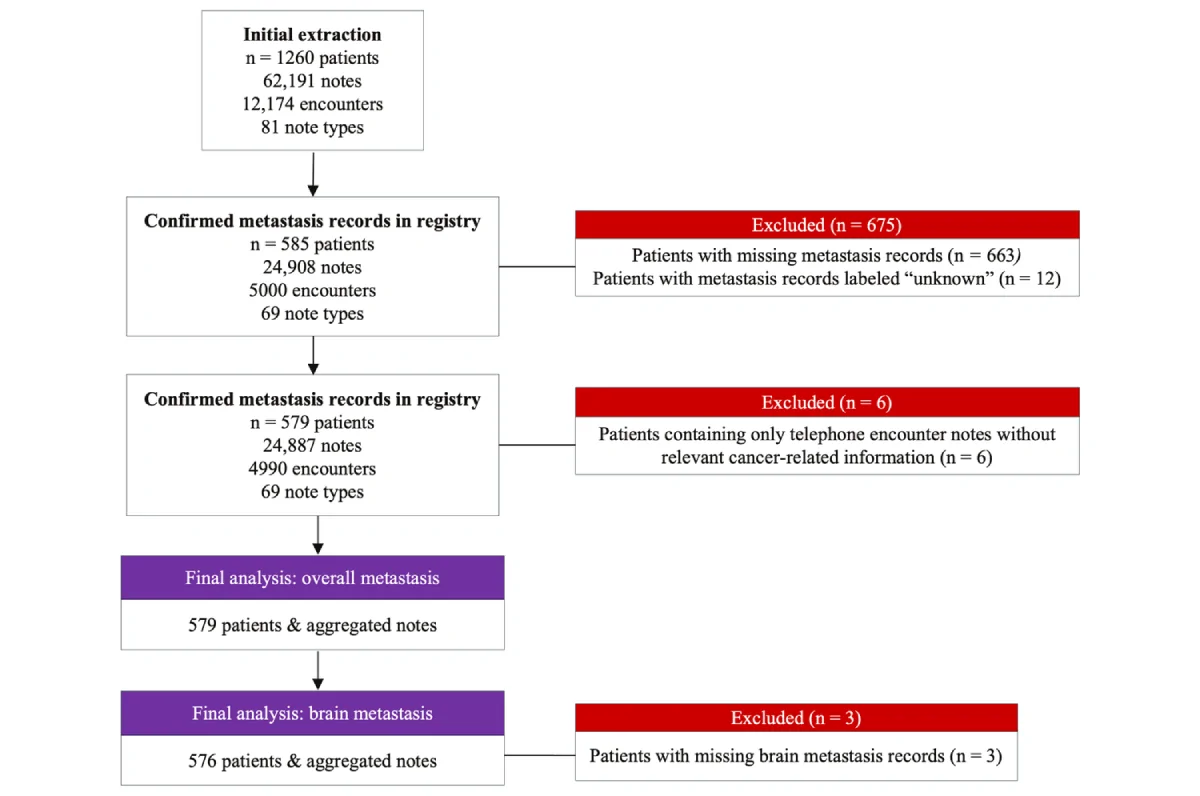
Flowchart of selection of cohort 1 following initial data extraction.

**Table 1:** Cohort 1 demographic characteristics.

		Total patients identified (n=1260)	Patients included in analysis (n=579)
Age at diagnosis (years), median (IQR)		72 (65-79)	73 (66-79)
Male, n (%)		670 (53.2)	273 (47.1)
Race, n (%)			
	White	599 (47.5)	279 (48.2)
	Black or African American	516 (40.9)	220 (38)
	Asian	72 (5.7)	37 (6.4)
	Other	20 (1.7)	13 (2.2)
	Unknown	53 (4.2)	30 (5.2)
Ethnicity, n (%)			
	Hispanic	27 (2.1)	14 (2.4)
	Non-Hispanic	1147 (91)	521 (90)
	Unknown	86 (6.8)	44 (7.6)

##### Reference Standard

We used the Winship Cancer Registry as our reference standard, which reports site-specific metastasis (liver, brain, bone, lung, distant lymph nodes, and other distant sites). Using these fields, we created our binary outcome label to identify overall metastasis. This label, encompassing all metastasis sites, was derived as follows: patients with at least one site-specific positive metastasis record were classified as metastasis-positive; patients with all-negative records were classified as metastasis-negative, and patients with at least one missing (n=663) or unknown (n=12) field, in the absence of a confirmed positive, were classified as metastasis unknown and excluded from model development. The registry brain metastasis field was used directly as the reference standard for brain metastasis analysis.

##### Note Inclusion Window

Because registry metastasis fields primarily capture disease status at initial diagnosis, clinical note inclusion was restricted to a window spanning 30 days before through 30 days after each patient’s formal cancer diagnosis date. This window maximized temporal correspondence between notes and registry labels. All note types within this window were eligible, including radiology reports, discharge summaries, progress notes, consultation notes, provider communication, nursing documentation, and other note types. The full distribution of notes across all note types is detailed in Table S1 in Multimedia Appendix 1.

#### Cohort 2: Independent Temporal Validation Cohort

To assess model generalizability across an earlier documentation period, we assembled an independent cohort of 22 patients with NSCLC whose records were extracted retrospectively from 2 completed randomized clinical trials conducted between 2010 and 2021. Trial enrollment required systematic imaging, resulting in dense radiology documentation in the historical records of these patients. A total of 644 radiology notes, including computed tomography, magnetic resonance imaging (MRI), X-ray, and other imaging reports, were extracted. Notes were not restricted to a diagnosis-adjacent window, and registry records were not used as the reference standard, since they did not capture documented metastatic progression. Manual note-level annotations served as the reference standard labels for this cohort (see ”Manual Annotation of Cohort 2” section). All models were applied to this cohort without retraining to serve as further external validation. Characteristics of both cohorts are provided in Table 2.

**Table 2:** Overall cohort characteristics.

		Cohort 1: primary registry-linked	Cohort 2: temporal validation	
Reference standard		Cancer registry	Manual annotation	
Documentation period		2023-2025	2010-2021	
Total patients with NSCLC^a^ identified, n		1260	22	
Patients with missing/unknown registry metastasis records, n (%)		675 (54)	0 (0)	
Patients included in analysis, n				
	Overall	579	22	
	Brain/CNS^b^	576	22	
Clinical notes included in analysis, n		24,887	644	
Note types		Heterogeneous (69 types)	Radiology only	
Patients with overall metastasis, n (%)		239 (41)	22 (100)	
Patients with brain/CNS metastasis, n (%)		89 (15)	8 (36)	

^a^NSCLC: non–small cell lung cancer.

^b^CNS: central nervous system.

#### Missingness-Recovery Dataset

The 675 patients excluded from primary model development, due to at least one missing or unknown registry metastasis record, represented 54% of the initial cohort 1 extraction pool. A total of 12 of these patients had unknown brain metastasis records. Notes from these patients were retained for a downstream exploratory analysis. This dataset was used solely to assess whether our methods could recover structured metastasis information absent from registry fields. No reference standard was available for this group, and model predictions were qualitatively reviewed by selecting a small random sample of patients and manually assessing their clinical notes, which were aggregated to the patient level. (see “Exploratory Downstream Missingness-Recovery Analysis” section). This analysis is reported to describe the feasibility of our methods and not as a primary performance evaluation.

### Clinical Note Preprocessing

Prior to model input, all clinical notes were preprocessed through a keyword-based filtering pipeline to reduce noise while retaining clinically relevant content. Keywords were drawn from the publicly available Clinical Event Recognizer (CLEVER) dictionary, which contains clinical synonyms for oncology-relevant terms, supplemented by custom oncology-specific terms developed by the study team [30]. Representative CLEVER terms included “metastasis,” “widespread disease,” and “stage IV,” among other words and phrases. Custom additions included “high grade” and “malignant mass,” in addition to other words. All keywords used for preprocessing are available in Table S2 in Multimedia Appendix 1. For notes containing at least one keyword match, only sentences containing keywords were retained. For notes without any match, the full note text was preserved. This step was applied strictly as a noise-reduction measure to eliminate sentences irrelevant to the patient’s disease course. In cohort 1, all notes from each patient were aggregated at the patient level before preprocessing and model input, consistent with the patient-level registry reference standard. In cohort 2, notes were processed and input individually.

### Manual Annotation of Cohort 2

Two trained abstractors, both of whom have a background in public health and biomedical informatics, independently annotated radiology notes from cohort 2 under the supervision of a board-certified oncologist. A standardized schema targeted 2 binary note-level labels: (1) overall metastasis present vs absent and (2) CNS metastasis present vs absent. Annotators were instructed to label both explicit evidence (eg, “brain metastases confirmed on MRI”) and contextually implied evidence strongly suggestive of current metastatic disease, such as imaging findings or treatment references indicating metastatic involvement.

Annotators were provided with two forms of each note: (1) the preprocessed text on which the label was assigned, and (2) the complete original note available for additional context. A predefined annotation schema was used to assign two binary note-level labels: (1) overall metastasis present vs absent, and (2) CNS metastasis present vs absent (Supplemental Material 1 in Multimedia Appendix 1). Disagreements were resolved by discussion or oncologist adjudication. Annotation proceeded in 4 iterative rounds of increasing size (n=10, 100, 200, and 334 notes), with interannotator agreement assessed and discrepancies reconciled after each round. Final agreement, quantified by Cohen κ values across all rounds of annotation, was 0.93 for overall metastasis and 0.88 for CNS metastasis, indicating high agreement (Table 3). Final labels were assigned based on unanimous consensus or oncologist suggestion.

Annotators reviewed each note in full before assigning a label. Explicit evidence of metastasis presence was often contained within a single sentence such as “hepatic lesions consistent with metastatic spread,” “brain metastases confirmed on MRI,” or “malignant pleural effusion,” and one such statement was sufficient for a positive label. A metastasis negative label could be determined either by explicit evidence of a metastasis-negative test result or by absence of metastasis-positive language in the note. Metastasis negative labels therefore required review of the complete note. Annotators were also instructed to label positive imaging findings or treatment references indicating metastatic involvement as metastasis positive. Findings in which the documenting clinician expressed uncertainty, such as a lesion described as suspicious or worrisome for metastasis without confirmation, were labeled negative. Because evidence of metastasis was distributed across multiple phrases within a note, and because the negative class required complete review of the note to establish absence of metastasis-positive language, annotators did not mark individual text spans and instead assigned note-level labels based on the full note.

**Table 3:** Interannotator agreement for cohort 2.

Annotation round	Overall metastasis, Cohen κ	CNS^a^ metastasis, Cohen κ
Round 1 (n=10)	1	1
Round 2 (n=100)	0.92	0.80
Round 3 (n=200)	0.92	0.85
Round 4 (n=334)	0.93	0.91

^a^CNS: central nervous system.

### Model Development and Inference

We evaluated three pretrained LLMs representing distinct architectures: (1) GatorTron-base, (2) MedGemma-27B-text-it, and (3) Llama 3.1-70B [31-33]. Selection was restricted to open-weight models so that all inference could be performed within our institutional Health Insurance Portability and Accountability Act (HIPAA)–secure environment without transmitting protected health information externally, allowing for reproducibility at other institutions. Within this constraint, models were chosen to represent the approaches currently used for clinical extraction: a clinically pretrained encoder applied with supervised fine-tuning, a general-domain instruction-tuned generative model applied zero-shot, and a medically pretrained model of the same generative type.

GatorTron is a transformer encoder pretrained on more than 90 billion words of deidentified clinical text, biomedical literature, and other English text [31]. Encoder architectures are well suited to sequence classification using real-world EHR data, and our registry-derived metastasis fields provided labels sufficient for supervised fine-tuning [34]. Additionally, a comparative analysis of pretrained biomedical models reported that GatorTron’s ability to learn nuanced clinical information within unstructured clinical text makes it suitable for clinical extraction tasks, compared to other BERT-based models [35]. Prior published comparisons have also demonstrated comparable performance between the GatorTron-base and GatorTron-large models. He et al [36] evaluated both GatorTron-base and GatorTron-large on clinical temporal relation extraction, finding that GatorTron-base with hard prompting achieved the highest micro *F*_1_-score (89.54%), exceeding GatorTron-large (88.06%). Standard fine-tuning of GatorTron-base achieved a micro *F*_1_-score of 85.95% [36]. Peng et al [37] similarly reported strong performance of GatorTron-base across tasks, including clinical concept extraction and medical relation extraction, achieving *F*_1_-scores of 0.89 and 0.96, respectively, on the 2018 n2c2 dataset [38]. Given this comparable performance and the lower computational cost required for deployment, GatorTron-base was selected for this study.

We began by benchmarking the non–fine-tuned GatorTron-base model (345M parameters) for binary classification of overall metastasis on a subset of cohort 1. This initial benchmarking yielded random chance predictions (*F*_1_-score=0.46), indicating a need for further fine-tuning on our institutional cohort [31]. We fine-tuned 2 separate GatorTron-base models for binary classification of overall metastasis (train: n=463, 80%; test: n=116, 20%) and brain/CNS metastasis (train: n=461, 80%; test: n=115, 20%). To mitigate label noise arising from ambiguous phrasing in clinical note text, training incorporated extraction of key linguistic features in the clinical text. First, a list of metastasis-relevant terms, from the CLEVER dictionary, was used to capture mentions of metastasis presence. For fine-tuning of brain/CNS metastasis classification, these terms were expanded to include brain/CNS site-specific verbiage. Next, NegEx-based negation detection was implemented to handle negated metastasis mentions [38]. Finally, a curated list of uncertainty expressions (eg, “suspicious for metastasis” and “may represent metastasis”) was implemented to handle unconfirmed metastasis mentions. These features were encoded as binary indicators.

Notes in the 80% train set were further partitioned into an 80% train set (overall metastasis: n=370; brain/CNS metastasis: n=369) and 20% validation set (overall metastasis: n=93; brain/CNS metastasis: n=92), stratified based on the binary outcome label. Since clinical notes were aggregated at the patient level prior to preprocessing, the unit of analysis was the aggregated patient document. Following preprocessing, cohort 1 documents averaged 7082.3 tokens in length (median 3382, IQR 1179–8795; range 12-87,293). Summary statistics of note lengths per cohort and average number of notes per patient are provided in Tables S3 and S4 in Multimedia Appendix 1. Patients were first divided into a model training set (80%) and a held-out test set (20%: overall metastasis n=116, brain/CNS metastasis n=115). The training set was then partitioned into training (80%: overall metastasis n=370; brain/CNS metastasis n=369) and validation (20%: overall metastasis n=93; brain/CNS metastasis n=92) subsets, with all splits stratified on the binary outcome label. GatorTron accepts a maximum input of 512 tokens, so documents were tokenized and segmented into overlapping 512-token chunks with a stride of 128 tokens, yielding a mean of approximately 19 chunks per patient document.

We trained a custom classifier head that concatenated the 3 linguistic features, described above, into a single feature vector. This feature vector, along with the text representation generated by GatorTron, was input into a feedforward neural network, which incorporated contextual and rule-based information to generate a binary prediction of metastasis presence. The 20% validation set was used to identify the optimal probability threshold for metastasis classification by evaluating thresholds between 0 and 1 and selecting the probability value that optimized the *F*_1_-score per patient. Final inference was conducted on the 20% hold-out test set (overall metastasis: n=116; brain/CNS metastasis: n=115). Final patient-level predictions were assigned based on the maximum probability predicted across chunks corresponding to each patient’s note. Predictions on cohort 2 were generated using the final fine-tuned model without further retraining. Probability thresholds were optimized on the cohort 1 validation set and applied directly to cohort 2. Optimizing thresholds on cohort 2 would have required its outcome labels and would therefore no longer constitute independent validation. Applying the cohort 1 threshold maintained a consistent decision rule across cohorts and allowed evaluation of whether the approach transfers to an independent cohort with different note characteristics without cohort-specific fine-tuning.

MedGemma-27B-text-it is a biomedical variant of Google’s Gemma model, optimized for medical text and image understanding [32]. Prior literature has demonstrated MedGemma’s ability to perform clinical information extraction from unstructured medical notes using prompt-based strategies, supporting its application in clinical information extraction tasks [39]. However, given MedGemma’s recent release at the time of this study, there was limited evidence of its use in oncology notes for cancer-related information extraction, reflecting a knowledge gap that this study sought to address. We evaluated the 27 billion parameter instruction-tuned text model in a zero-shot setting using structured prompts that instructed the model to classify whether the provided note text documented the target condition and to return a constrained binary response (“Metastasis: YES” or “Metastasis: NO”). Greedy decoding was used, in which stochastic sampling was disabled (do_sample=False), such that the highest probability token was selected at each generation step. A fixed maximum output length was imposed to constrain the models’ responses. Prompt templates are provided in Supplemental Materials 2 and 3 in Multimedia Appendix 1.

Llama 3.1-70B-Instruct is a general-domain open-source LLM developed by Meta, trained on a large-scale corpus of general text and optimized for instruction-following [40]. Given that the prior 2 models were pretrained on biomedical data, evaluation of the Llama model allows for an understanding of the capability of general-domain models to comprehend dense clinical text. Furthermore, the Llama model has previously been shown to outperform BERT-based models and successfully conduct structured extraction tasks from clinical notes [23]. Therefore, its evaluation in this study, alongside GatorTron and MedGemma, may challenge current notions that encoder biomedical models are superior for clinical extraction tasks [23]. Llama3 was evaluated using an identical zero-shot prompt structure to MedGemma, decoding configuration, and constrained binary output format as MedGemma for both extraction tasks. Because the two are similar in architecture and prompting strategy and differ primarily in their pretraining, their comparison is informative about whether medical-domain pretraining is associated with better performance on this task.

Since the encoder model was fine-tuned while the generative models were prompted zero-shot, this study is an evaluation of distinct NLP approaches for metastasis classification rather than a direct comparison of model architectures. A pipeline depicting an overview of note preprocessing and inference is provided in Figure 2. All model weights were obtained from Hugging Face and deployed locally on an institutional, HIPAA-secure computing infrastructure [41-43]. All analyses were implemented in Python 3.10. All code is available on GitHub [44]. Models were run on a high-performance cluster with an AMD EPYC 9254 Processor (24 CPU cores/48 CPU threads), 348 GB RAM, and 2 NVIDIA RTX 6000 Ada Generation GPUs (48 GB VRAM per GPU), and 60 TB of NVMe-backed local scratch space in /tmp. The computational job used to run these models was allocated 2 CPU cores, 128 GB RAM, and 2 GPUs. Training time for the GatorTron model took 32 minutes on the training cohort (n=463). Inference time for all 3 models was approximately 4.5 minutes per model on the test sets (n=116), averaging approximately 2.3 seconds per note.

**Figure 2:**
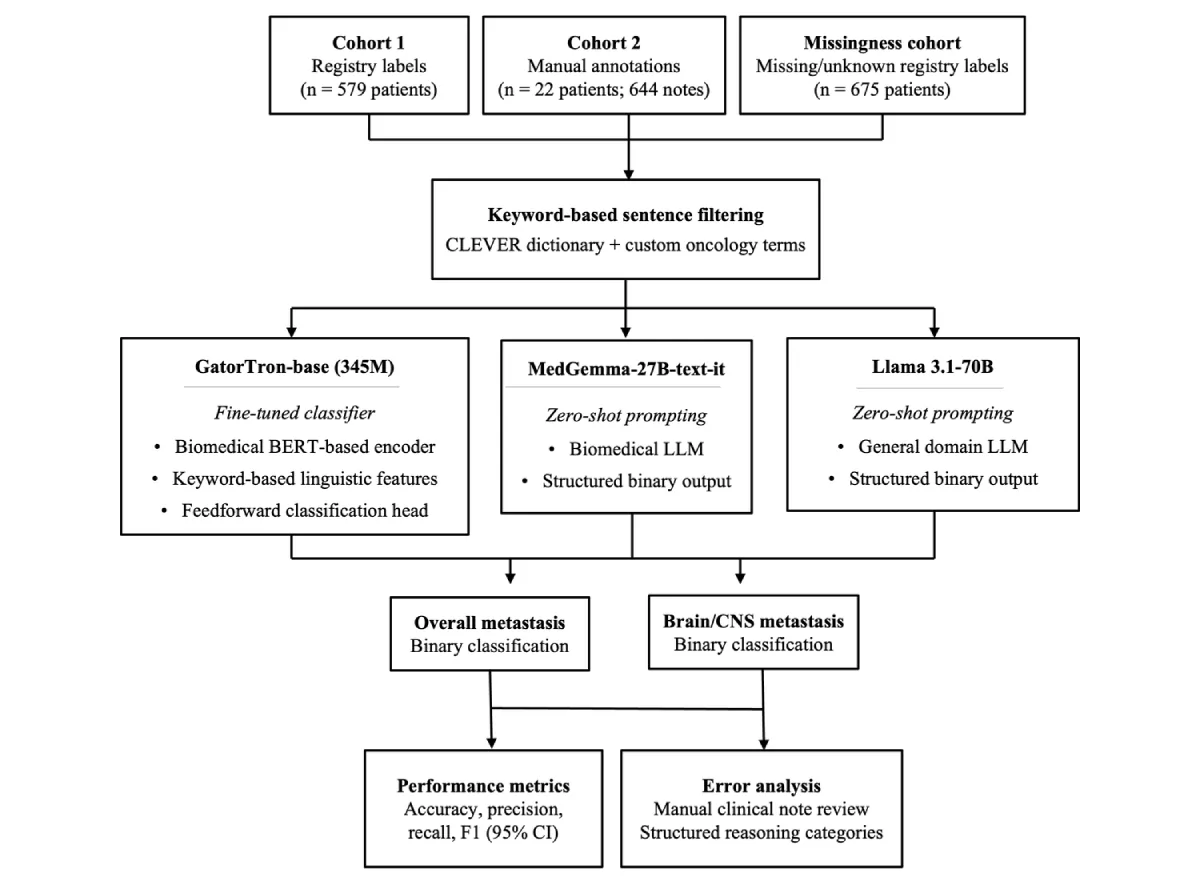
Note preprocessing and model inference pipeline. CLEVER: Clinical Event Recognizer; CNS: central nervous system; LLM: large language model.

### Data Partitioning and Model Evaluation

GatorTron fine-tuning and testing were conducted on cohort 1. MedGemma and Llama3 did not undergo fine-tuning and were instead evaluated under a zero-shot prompt setting. Cohort 1 evaluations for all models were conducted on the 20% test set used in the evaluation of the fine-tuned GatorTron model (overall metastasis: n=116; brain metastasis: n=115).

Cohort 2 served as an independent, out-of-sample test set for all 3 models. Since note-level manual annotations served as the reference standard for cohort 2, model evaluation was conducted at both the note-level (n=644) and the patient-level (n=22). Patient-level reference standards were derived by aggregating note-level annotations, such that if any note of a patient is labeled metastasis positive, the patient-level label is metastasis positive. For each model, cohort, and prediction level, we computed accuracy, precision, recall, and *F*_1_-score. The 95% CIs were estimated using bootstrapped resampling with 10,000 iterations. All metrics were reported separately for the overall metastasis and brain/CNS metastasis tasks.

Furthermore, to assess whether our methods extend beyond aggregated metastasis status, we additionally evaluated our best-performing model on 3 site-specific classification tasks based on available metastasis fields in the cancer registry: bone, liver, and distant lymph node metastasis. The evaluation used the same held-out test set (n=116).

### Error Analysis

For the best and poorest performing models on each extraction task in the development cohort, all misclassified cases were examined qualitatively. Clinical notes from each error were manually reviewed and assigned to one of four prespecified error categories: (1) incorrect model prediction despite obvious note content; (2) ambiguous clinical text, in which metastatic status was uncertain or unconfirmed by imaging, laboratory testing, or biopsy; (3) correct model prediction with an erroneous registry label; or (4) missing clinical text, in which relevant documentation was likely absent from the available note. Category definitions and representative examples are provided in Table 4.

**Table 4:** Structured reasoning categories used in qualitative analysis of prediction errors.

Error category	Description	Example
Incorrect prediction	The model assigned an incorrect prediction despite clear indication of positive or negative metastasis presence in the clinical note text.	“Suspected metastasis confirmed to be benign meningioma.” In this case, the note explicitly confirms negative metastasis, but the model predicts a positive case.
Ambiguous text	The clinical note text has unclear confirmation of metastasis presence. The note expresses uncertainty of metastasis presence without documented confirmation via imaging, laboratory test, or biopsy. The clinical note text may also indicate suspicion of an additional primary cancer, which the model predicts as metastasis.	“Brain metastasis cannot be ruled out.” “There are several malignant nodular lesions.” If no imaging, laboratory test, or biopsy confirmation is documented, the model may predict the incorrect label.
Correct prediction and incorrect registry label	In some cases of false positive predictions, the available clinical note demonstrates clear mentions of metastasis presence, either indicating an error in the registry label or an update in the patient’s cancer diagnosis not reflected in the registry.	“Biopsy results confirm osseous mets.” If this confirmation is documented around the time of initial cancer diagnosis but is not reflected in the cancer registry, this may reflect an error in the registry label.
Missing clinical text	In some cases of false negative predictions, the available clinical note does not clearly state any mention of metastasis. Therefore, rather than a failure of the model, these errors indicate the possible presence of additional notes, unavailable to us, which may have included metastasis information.	If the registry indicates metastasis to a specific site, but there is no language in the clinical note relevant to metastasis, metastasis testing, or even mention of the site, the model prediction will not align with the registry label.

### Exploratory Downstream Missingness-Recovery Analysis

The best-performing model for each extraction task was applied to the 675 patients in the missingness-recovery dataset. This analysis was conducted to evaluate the feasibility of leveraging our models to recover metastasis information from narrative text. To assess predictive performance, random samples of 10 predicted-positive and 10 predicted-negative cases were drawn. Their notes were manually reviewed and labeled prior to viewing model predictions to ensure unbiased assessment. Concordance between manual labels and model predictions was then evaluated. Given the small validation sample, findings are exploratory rather than definitive of our model performance.

### Ethics Approval

This study was approved by the Emory University Institutional Review Board (protocol 2025P010922) under the expedited review process, with a complete waiver of informed consent and HIPAA authorization.

## Results

### Cohort Characteristics

Among 1260 patients with NSCLC identified from the Winship Cancer Registry and linked to clinical notes, restricted to analytic cases diagnosed and/or treated at Emory, metastasis status was missing or recorded as unknown in 54% (675/1260) of patients. Cohort 1 included 579 patients with complete registry metastasis data and 24,898 clinical notes; 41% (239/579) had metastasis at any site, and 15% (89/579) had brain metastasis at diagnosis. Cohort 2 included 22 patients and 644 radiology notes. All patients had metastasis at any site, and 36% (8/22) had CNS metastasis. At the note level, 51% (328/644) of notes were positive for metastasis and 11% (71/644) for CNS metastasis. Keyword-based preprocessing reduced mean document length by approximately 71% in both cohorts, from 24,193 to 7082 tokens per aggregated patient note in cohort 1 and from 364 to 107 tokens per note in cohort 2 (Table S3 in Multimedia Appendix 1).

### Model Performance: Overall Metastasis Extraction

All models demonstrated strong performance (Table 5). In cohort 1, MedGemma achieved the highest *F*_1_-score (0.80), followed by Llama3 (*F*_1_-score=0.76) and fine-tuned GatorTron (*F*_1_-score=0.72). In cohort 2, all models achieved high patient-level scores, which was expected given the dense radiological documentation and the presence of metastasis in all patients. At the note level, MedGemma achieved the highest *F*_1_-score (0.93), followed by Llama3 (*F*_1_-score=0.88) and GatorTron (*F*_1_-score=0.76). The optimal probability threshold for overall metastasis classification was 0.58.

**Table 5:** Performance metrics and 95% CIs for overall metastasis prediction.

Model and cohort			Prediction, n		Accuracy (95% CI)		Precision (95% CI)	Recall (95% CI)		*F*_1_-score (95% CI)
GatorTron-base (fine-tuned)										
	Cohort 1 (primary development)	116^a^		0.74 (0.66-0.83)		0.71 (0.65-0.81)		0.74 (0.65-0.84)	0.72 (0.63-0.80)	
	Cohort 2	22^a^		0.95 (0.86-1.0)		1.0 (1.0-1.0)		0.95 (0.86-1.0)	0.98 (0.93-1.0)	
	Cohort 2	644^b^		0.71 (0.67-0.74)		0.65 (0.61-0.70)		0.91 (0.88-0.94)	0.76 (0.73-0.79)	
Llama 3.1-70B-Instruct										
	Cohort 1	116^a^		0.76 (0.68-0.84)		0.69 (0.58-0.80)		0.85 (0.74-0.94)	0.76 (0.67-0.84)	
	Cohort 2	22^a^		1.0 (1.0-1.0)		1.0 (1.0-1.0)		1.0 (1.0-1.0)	1.0 (1.0-1.0)	
	Cohort 2	644^b^		0.87 (0.85-0.90)		0.84 (0.80-0.88)		0.92 (0.89-0.95)	0.88 (0.85-0.91)	
MedGemma-27B-text-it										
	Cohort 1	116^a^		0.80 (0.72-0.87)		0.75 (0.64-0.86)		0.85 (0.76-0.94)	0.80 (0.72-0.87)	
	Cohort 2	22^a^		1.0 (1.0-1.0)		1.0 (1.0-1.0)		1.0 (1.0-1.0)	1.0 (1.0-1.0)	
	Cohort 2	644^b^		0.93 (0.91-0.95)		0.97 (0.95-0.99)		0.88 (0.85-0.92)	0.93 (0.90-0.95)	

^a^Patient-level.

^b^Note-level.

### Model Performance: Brain/CNS Metastasis Extraction

In cohort 1, Llama3 achieved the highest *F*_1_-score (0.79), followed by MedGemma (*F*_1_-score=0.76) and GatorTron (*F*_1_-score=0.50). In cohort 2, Llama3 achieved the highest *F*_1_-scores (patient-level *F*_1_-score=0.93; note-level *F*_1_-score=0.86). MedGemma achieved comparable note-level performance (*F*_1_-score=0.86) with a lower patient-level score (*F*_1_-score=0.70). GatorTron achieved a patient-level *F*_1_-score of 0.72 and a note-level *F*_1_-score of 0.50. The optimal probability threshold for brain/CNS metastasis classification was 0.40. Performance metrics for brain/CNS metastasis are provided in Table 6.

**Table 6:** Performance metrics and 95% CIs for brain/central nervous system metastasis prediction.

Model and cohort		Prediction, n	Accuracy (95% CI)	Precision (95% CI)	Recall (95% CI)	*F*_1_-score (95% CI)
GatorTron-base (fine-tuned)						
	Cohort 1 (primary development)	115^a^	0.88 (0.80-0.96)	0.50 (0.38-0.64)	0.50 (0.26-0.71)	0.50 (0.34-0.66)
	Cohort 2	22^a^	0.73 (0.55-0.91)	0.57 (0.30-0.82)	1.0 (1.0-1.0)	0.72 (0.46-0.90)
	Cohort 2	644^b^	0.81 (0.78-0.84)	0.35 (0.28-0.42)	0.83 (0.75-0.92)	0.50 (0.42-0.67)
Llama 3.1-70B-Instruct						
	Cohort 1	115^a^	0.96 (0.91-0.99)	0.91 (0.69-1.0)	0.71 (0.46-0.94)	0.79 (0.59-0.96)
	Cohort 2	22^a^	0.95 (0.86-1.0)	0.88 (0.62-1.0)	1.0 (1.0-1.0)	0.93 (0.77-1.0)
	Cohort 2	644^b^	0.96 (0.95-0.98)	0.82 (0.73-0.90)	0.90 (0.82-0.97)	0.86 (0.79-0.91)
MedGemma-27B-text-it						
	Cohort 1	115^a^	0.95 (0.90-0.98)	0.83 (0.58-1.0)	0.72 (0.45-0.94)	0.76 (0.54-0.92)
	Cohort 2	22^a^	0.82 (0.74-0.95)	0.83 (0.43-1.0)	0.63 (0.25-1.0)	0.70 (0.33-0.93)
	Cohort 2	644^b^	0.97 (0.95-0.98)	0.83 (0.75-0.92)	0.89 (0.81-0.96)	0.86 (0.79-0.91)

^a^Patient-level.

^b^Note-level.

### Error Analysis

Prediction errors from the best and poorest performing models for each classification task in the held-out test set of cohort 1 (overall metastasis n=116; brain/CNS metastasis n=115) were qualitatively analyzed and assigned to a category of reasoning. Corresponding predictions from the other 2 models were assessed in parallel.

#### Overall Metastasis Prediction

##### GatorTron

GatorTron generated 14 false negative predictions and 16 false positive predictions. Less than half of the errors generated (n=13) were due to incorrect model errors, all of which were predicted correctly by MedGemma and Llama3. The primary reason for other errors was ambiguous phrasing in the clinical text (n=10), in which clinical documentation indicated “suspicious,” “concerning,” or “probable” metastasis without clear confirmation through laboratory test results. Another reason was that the model correctly generated positive predictions (n=4), despite a metastasis-negative registry label. In these cases, patient notes explicitly mentioned a diagnosis of metastatic cancer, which was not reflected in the registry but identified by our model. Finally, multiple false negative cases (n=3) were due to missing clinical text. A detailed error analysis is provided in Table S6 in Multimedia Appendix 1.

##### MedGemma

For prediction of overall metastasis, MedGemma generated 8 false negative predictions and 15 false positive predictions. In 9 instances, the model generated correct positive predictions despite conflicting registry records. Errors also occurred due to missing clinical text (n=6) and ambiguous clinical text (n=6; 4 false positive and 2 false negative predictions). Finally, the model produced one false positive prediction and one false negative prediction, both categorized as incorrect and predicted correctly by Llama3 and GatorTron. A detailed error analysis is provided in Table S7 in Multimedia Appendix 1.

#### Brain/CNS Metastasis Prediction

##### GatorTron

For brain/CNS metastasis, GatorTron generated 7 false positive predictions and 7 false negative predictions. Most errors were caused by incorrect predictions (n=10), all of which were predicted correctly by MedGemma and Llama3. Other errors were due to missing text (n=3) and ambiguous text (n=1). A detailed error analysis is provided in Table S8 in Multimedia Appendix 1.

##### Llama3

Llama3 generated 4 false negative predictions due to missing clinical text and one false positive prediction, which was determined to be incorrect. The incorrect false positive prediction was predicted correctly by MedGemma and GatorTron. A detailed error analysis is provided in Table S9 in Multimedia Appendix 1.

### Site-Specific Metastasis Extraction

Since MedGemma was the best-performing model for overall metastasis, we further examined its site-specific metastasis classification performance (Table 7). Performance for bone metastasis (*F*_1_-score=0.79) and liver metastasis (*F*_1_-score=0.82) was comparable to that observed for overall metastasis classification. Performance for distant lymph node metastasis was lower (*F*_1_-score=0.29), reflecting high recall (0.87) with low precision (0.18) at a prevalence of 1.7%.

**Table 7:** Performance metrics and 95% CIs for site-specific metastasis prediction.

Model and metastasis site (n=116)		Prevalence^a^	Accuracy (95% CI)	Precision (95% CI)	Recall (95% CI)	*F*_1_-score (95% CI)
MedGemma-27B-text-it						
	Bone	0.16	0.93 (0.88-0.97)	0.79 (0.58-0.95)	0.79 (0.59-0.95)	0.79 (0.62-0.91)
	Liver	0.12	0.96 (0.91-0.99)	0.80 (0.57-1.0)	0.86 (0.64-1.0)	0.82 (0.64-0.96)
	Distant lymph nodes	0.02	0.92 (0.87-0.96)	0.18 (0.00-0.45)	1.0 (0.00-1.0)	0.31 (0.00-0.62)

^a^Proportion of test-set patients with registry-recorded metastasis at the specified site (n=116).

### Exploratory Missingness-Recovery Analysis

We evaluated whether models could recover metastasis information among 675 patients with at least one missing or unknown registry label (including 12 with unknown brain metastasis status). Using the best-performing model for overall metastasis, MedGemma classified 302 of 675 (44.7%) patients as metastasis-positive, closely resembling the prevalence of metastasis in cohort 1 (239/579, 41%). To assess validity, a random sample of 20 cases (10 predicted positive and 10 predicted negative) was manually annotated prior to viewing model results. Agreement with model predictions yielded accuracy=0.90 and *F*_1_-score=0.89. Two false positive errors were identified in the prediction of overall metastasis due to several ambiguous phrases regarding suspicion of metastasis without clear confirmation. For brain/CNS metastasis, all 12 patients with unknown registry status were manually annotated as negative and correctly classified by Llama3.

## Discussion

### Principal Findings

LLM-based methods demonstrated strong performance for extracting overall and CNS metastasis from real-world NSCLC clinical notes, with performance varying by task and evaluation setting. Additional exploratory analyses suggest that clinically meaningful information about metastasis can be recovered from narrative documentation when structured registry data are missing. These findings highlight a core limitation of current oncology data systems. Cancer registries, while essential, are temporally limited and incomplete, whereas clinical notes can provide longitudinal, detailed documentation of disease evolution. LLMs offer a practical mechanism to extract clinically relevant information within routine documentation, complementing structured data.

Earlier studies have largely focused on single cohorts or limited note types, particularly radiology reports and discharge summaries [24,25]. By leveraging a registry-linked cohort with heterogeneous oncology documentation, we provide a more realistic evaluation of real-world performance. Furthermore, few studies have evaluated and compared distinct NLP strategies, including clinically pretrained encoder models and large instruction-tuned generative models, on the same task [8,11,12]. In our study, baseline performance metrics demonstrated that the generative models (Llama3 and MedGemma) achieved higher performance in classifying both overall and brain/CNS metastasis presence, respectively, compared to the fine-tuned GatorTron model. Upon further inspection of model errors, generative models and fine-tuned encoder models appeared to serve complementary strengths, where certain prediction errors made by one model were correctly predicted by another. Additionally, while GatorTron was fine-tuned on clinical notes using linguistic features, Llama 3.1 and MedGemma were evaluated under zero-shot prompting strategies. Therefore, rather than a direct comparison of model architectures, this study showcases distinct LLM-based strategies for metastasis classification of clinical notes. CIs for several estimates overlap substantially, particularly for brain/CNS metastasis in cohort 1 and across tasks in cohort 2, and differences in reported scores should not be interpreted as establishing that one approach outperforms another.

Fine-tuned encoder models show promise for classifying metastasis presence and identifying potentially inaccurate registry records based on clinical note text; however, they may be sensitive to discrepancies present in their input data. Instruction-tuned generative models, even in a zero-shot prompt setting, demonstrate holistic understanding of clinical note text without this limitation, which may explain the differences in model performance in this study.

In this multicohort evaluation, we make 3 principal observations. First, metastasis information was substantially incomplete even within a cancer registry-linked dataset, with more than half of patients lacking definitive structured metastasis labels. Second, model performance varied, with the instruction-tuned generative models demonstrating holistic understanding of complex clinical language and the fine-tuned encoder model demonstrating strong classification performance but likely hindered by potentially inaccurate registry labels during training. Third, LLMs appear adept at correcting inaccurate registry records and recovering missing data.

Our first observation in this study is the high prevalence of missing data in structured metastasis labels, representing 54% (675/1260) of the patients identified in our initial extraction of cohort 1. This study extends prior clinical NLP work in important ways. While cancer registries serve as the gold standard for cancer surveillance, future studies may seek to use more curated real-world datasets for more robust model fine-tuning. Though inaccuracies and missingness in the cancer registry may have inhibited the fine-tuned encoder model from more accurately distinguishing between positive and negative metastasis cases, our model still demonstrated the ability to correct inaccurate data based on clinical text documentation. The use of metastasis labels from the cancer registry presents both an advantage and a challenge to fine-tuning our GatorTron model. Our cohort size (n=579 patients for overall metastasis; n=576 patients for brain/CNS metastasis) was comparable to prior work that fine-tuned the same model for metastasis extraction, which leveraged manually annotated computed tomography reports from 550 patients [45]. That work, however, manually annotated labels at the report level. Because registry metastasis fields are recorded at the patient level, each of our labels applied instead to an aggregated document (mean 7082, SD 9841 tokens) drawn from 69 distinct note types in which metastatic disease may be expressed in different forms. This heterogeneity better reflects real-world documentation but provides weaker supervision for a fine-tuned model, and it is intrinsic to registry-linked data. We did not evaluate performance as a function of training set size and therefore cannot determine whether the fine-tuned model was data-limited. The zero-shot models required no labeled data and were not subject to these constraints; their comparable or higher performance suggests that zero-shot approaches may be better suited to settings where only patient-level reference labels are available.

Our second observation in this study is that model performance patterns underscore that no single approach is uniformly optimal. Encoder models align well with structured labels; however, discrepancies in registry records may influence model outputs. In contrast, generative models appear more robust to heterogeneous, implicit, and context-dependent clinical language, which is particularly relevant for CNS metastasis, where documentation is often indirect and radiology-driven. This difference in performance may be attributed to the ability of generative models to better identify nuanced and complex site-specific language used in the documentation of brain/CNS without the influence of structured registry labels. Furthermore, GatorTron’s prediction of brain/CNS metastasis may have been inhibited by the class imbalance in brain/CNS metastasis cases during model fine-tuning, whereas this class imbalance was not impactful in a zero-shot prompting setting. These results support a use-case-specific deployment strategy rather than a single-model solution.

Our third observation in this study is that LLM-based extraction appears to be adept at correcting and recovering incomplete metastasis data in structured registry fields. Our error analysis indicated that many discrepancies were due to data limitations in reference labels or available clinical documentation rather than explicit model failure. Differences in registry labels compared to clinical documentation, missing clinical text, and ambiguity in clinical language accounted for most errors. In several cases, model predictions were clinically plausible despite conflicting with registry labels, underscoring the imperfect nature of structured ground truth in oncology datasets. Both the error analysis and exploratory missingness recovery analysis provide an initial signal that NLP may augment registry accuracy and completeness. The error analysis identified several positive metastasis cases that were not reflected in the registry labels, despite clinical documentation near the time of initial diagnosis. The exploratory missingness recovery analysis demonstrated high accuracy in recovering missing metastasis labels, based on manual review of a subset of notes. While preliminary, this suggests that clinically relevant signals embedded in narrative documentation can be systematically recovered. These findings have direct clinical and operational implications. NLP-based extraction can support cancer registry workflows by prioritizing high-yield cases and improving completeness. It may also enable more accurate cohort construction and clinical trial screening, where metastasis status, particularly CNS involvement, is a key eligibility criterion.

Our study contributes to the current field of clinical NLP in a few key ways. First, previous metastasis extraction studies have predominantly focused on single document types, namely radiology reports and discharge summaries, while our evaluation included 69 distinct note types [24,25,45]. The incorporation of heterogeneous clinical documentation more closely reflects the setting in which LLM-based cancer abstraction would be deployed. It also provides more robust evidence of each model’s ability to comprehend and extract structured data from complex, varied clinical language. Second, we evaluated not just distinct LLMs but distinct modeling approaches for metastasis extraction. Recent literature has begun to compare different LLM-based approaches for clinical note extraction, including encoder-only, decoder-only, and prompt-based instruction-tuned LLMs in zero-shot and few-shot settings [37,46]. Such comparisons have not previously been reported for metastasis extraction. Because models were evaluated using different adaptation strategies, this study does not permit a direct comparison of model architectures, but it does provide an evaluation of distinct LLM-based methodologies for clinical note extraction. Third, we used both registry data and human annotations as reference standards, in cohort 1 and cohort 2 respectively. This allowed model performance to be assessed against existing structured registry fields while independently evaluating it against human annotation. Metastasis status was missing or recorded as unknown for 54% (675/1260) of registry-linked patients in the source population, consistent with reports of incomplete and discordant registry documentation [47].

Our error analysis further indicated that many apparent model errors reflected gaps in the registry rather than misclassification, illustrating the limitations of registry-derived labels as a reference standard for model development. Finally, despite these inconsistencies, LLM-based approaches may support the recovery of missing or incorrect registry labels: in the exploratory recovery analysis, predictions for patients with missing registry values agreed with manual annotation at accuracy 0.90 and *F*_1_-score 0.89. Few studies have used LLM-based methods to assess the recovery of information missing from structured registry fields.

Our study has several limitations. First, cohort 1 (the primary development cohort) relied on cancer registry labels that contain limitations, including accuracy of label and inability to capture metastatic progression. Thus, discordance between registry labels and model predictions does not necessarily indicate model error, since the reference standard itself may be imperfect. Furthermore, there is a possibility that, though documentation spanned one month before and one month after initial diagnosis, metastasis cases diagnosed within one month after initial cancer diagnosis were not updated in the registry.

Second, cohort 2 was small and derived from clinical trial populations with dense radiologic follow-up, which may limit generalizability to routine care settings. All models demonstrated higher performance in cohort 2, likely due to the minimal heterogeneity of note types. Radiology notes are likely to contain dense documentation of metastatic disease presence, which may have contributed to improved model performance in cohort 2. Moreover, all patients in cohort 2 had overall metastasis, so patient-level metrics for that task do not reflect the models’ ability to distinguish metastasis-positive from metastasis-negative patients. The note-level evaluation is therefore the more informative measure of discriminative performance in this cohort, and patient-level results should be interpreted as complementary rather than as an indication of the model’s discriminative ability. Note-level metrics may, themselves, be optimistic, since each patient contributed multiple notes that often contained similar or repeated phrasing and are therefore not independent observations.

Third, the performance metrics of cohort 2 require further contextualization, given its small cohort size and class imbalance in the patient-level evaluation. Since all patients were found to have metastasis, the patient-level performance metrics do not demonstrate the models’ ability to distinguish between metastasis-positive and metastasis-negative patients. Additionally, the note-level performance metrics may reflect a biased overestimation of the models’ ability to classify metastasis presence. Each patient contributed multiple notes, which may have contained similar or repeated phrasing, likely leading the model to assign identical metastasis predictions for multiple notes based on similar language seen across notes.

Fourth, the use of different reference standards across cohorts (registry-based at the patient level in cohort 1 vs manual note-level annotation in cohort 2) complicates direct comparison, particularly given differences in temporal scope and definition of CNS metastasis. Certain prediction errors made by the models on cohort 1 may have been a result of incorrect registry labels. Since the ground truth label was more reliable in cohort 2 notes, model performance may have appeared to be better in this cohort.

Fifth, models of differing underlying architectures were evaluated in distinct ways, wherein GatorTron was fine-tuned, while generative models were evaluated in a zero-shot setting. Additionally, encoder models with extended context windows, compared to GatorTron, such as those built on the ModernBERT architecture, have since become available and can process inputs of up to 8192 tokens. Such models would accommodate most documents in this cohort without the chunking and max-pooling strategies used in our study. Evaluating whether long-context modeling improves performance of metastasis classification would be a useful extension of this work. The difference in computational requirements across the 3 models is a crucial consideration for deployment of these models in real-world clinical settings. The Llama 3.1 70-billion-parameter model has larger memory requirements compared to the GatorTron 345-million-parameter model, and this difference should be considered when inference is run on constrained hardware. Model selection in real-world clinical settings should consider this trade-off between performance and computational requirements.

Sixth, the keyword-based preprocessing strategy may have excluded relevant contextual information, potentially limiting sensitivity to implicitly expressed metastasis. Seventh, the study was conducted at a single institution, and broader external validation is needed. Finally, the missing recovery analysis was exploratory and based on a small sample. A larger-scale validation should be conducted to further evaluate the potential of recovering missing metastasis records. Similarly, site-specific evaluation was informative for bone and liver metastasis but not for distant lymph node metastasis, where prevalence in the test set was 1.7%, and the resulting estimates were unstable. Our estimates demonstrated a high presence of false positive predictions, so future evaluations of site-specific metastasis at infrequently documented sites would require a much larger test set.

Several factors affect the generalizability of our findings. Both patient cohorts were specific to one health care institution, and all patients were diagnosed with NSCLC. These constraints pose a challenge in assessing the models’ generalizability to cohorts at other institutions and with other cancer types, and clinical terminologies and documentation practices may differ elsewhere. Our study design does, however, offer 2 features relevant to interpretation. First, the variation in clinical note types allows the model evaluations to be more representative of real-world EHR documentation. Second, the use of 2 cohorts from different documentation periods, with different reference standards, provided a second evaluation setting and a preliminary view of model performance across distinct clinical language and documentation styles. Cohort 2 was small (n=22), drawn from a clinical trial population with dense radiologic follow-up, and comprised entirely of metastasis-positive patients, so it does not by itself establish generalizability of our methods. It provides a second evaluation setting with an independent reference standard and a different documentation period, but its size and composition prevent its results from generalizing to routine care. Additionally, the patient-level estimates in this cohort carry wide CIs. Similarly, the missingness-recovery analysis was validated against manual annotation of 20 cases, demonstrating the feasibility of our methods but not sufficient to estimate recovery performance with precision. Therefore, the results of the error analysis should be regarded as preliminary. Both findings require confirmation in larger and more diverse cohorts in order to demonstrate the reliability of these methods in other settings.

### Conclusion

In this study, the instruction-tuned generative models, evaluated in a zero-shot setting, performed best at classifying metastasis. The high degree of missing metastasis data, even within a cancer registry-linked cohort, underscores a key limitation of structured oncology data and highlights the value of note-based ascertainment. Exploratory analyses further suggest that metastasis information documented in clinical narratives can be partially recovered when structured fields are incomplete. These findings support the use of NLP to complement cancer registry data for metastasis ascertainment, cohort identification, and trial screening, while requiring further validation before broader use.

## Appendix

Multimedia Appendix 1 Note distributions, preprocessing and feature extraction keywords, metastasis annotation guidelines, large language model prompt templates, and model error analyses.

## Abbreviations

CLEVER: Clinical Event Recognizer
CNS: central nervous system
EHR: electronic health record
HIPAA: Health Insurance Portability and Accountability Act
LLM: large language model
ML: machine learning
MRI: magnetic resonance imaging
NLP: natural language processing
NSCLC: non–small cell lung cancer

## Data Availability

The datasets analyzed in this study are not publicly available due to institutional policies regarding Health Insurance Portability and Accountability Act (HIPAA)–protected medical files.
